# MRI of the lung (1/3): methods

**DOI:** 10.1007/s13244-012-0176-x

**Published:** 2012-06-13

**Authors:** J. M. Wild, H. Marshall, M. Bock, L. R. Schad, P. M. Jakob, M. Puderbach, F. Molinari, E. J. R. Van Beek, J. Biederer

**Affiliations:** 1 Academic Radiology, Royal Hallamshire Hospital Sheffield, University of Sheffield, Sheffield, S10 2JF UK; 2 Radiology—Medical Physics, University Hospital Freiburg, Breisacher Straße 60a, 79106 Freiburg, Germany; 3 Computer Assisted Clinical Medicine, Heidelberg University, Theodor-Kutzer-Ufer 1-3, 68167 Mannheim, Germany; 4 Department of Experimental Physics 5, University of Würzburg, Am Hubland, 97074 Würzburg, Germany; 5 Department of Radiology, German Cancer Research Center (DKFZ), Im Neuenheimer Feld 280, 69120 Heidelberg, Germany; 6 Department of Diagnostic and Interventional Radiology, Chest Clinics at University Hospital Heidelberg, Amalienstraße 5, 69126 Heidelberg, Germany; 7 Department of Radiology, C. H. R. U. Lille, Hôpital Calmette, Boulevard du Prof. J. Leclercq, 59037 Lille, France; 8 Clinical Research Imaging Centre, University of Edinburgh, 47 Little France Crescent, Edinburgh, EH16 4TJ UK; 9 Department of Diagnostic Radiology, University Hospital Schleswig-Holstein, Campus Kiel, Arnold-Heller-Straße 3, Haus 23, 24105 Kiel, Germany

**Keywords:** MRI, Lung, Proton

## Abstract

Proton magnetic resonance imaging (MRI) has recently emerged as a clinical tool to image the lungs. This paper outlines the current technical aspects of MRI pulse sequences, radiofrequency (RF) coils and MRI system requirements needed for imaging the pulmonary parenchyma and vasculature. Lung MRI techniques are presented as a “technical toolkit”, from which MR protocols will be composed in the subsequent papers for comprehensive imaging of lung disease and function (parts 2 and 3). This paper is pitched at MR scientists, technicians and radiologists who are interested in understanding and establishing lung MRI methods. Images from a 1.5 T scanner are used for illustration of the sequences and methods that are highlighted.

*Main Messages*

• *Outline of the hardware and pulse sequence requirements for proton lung MRI*

• *Overview of pulse sequences for lung parenchyma, vascular and functional imaging with protons*

• *Demonstration of the pulse-sequence building blocks for clinical lung MRI protocols*

## Introduction

Proton magnetic resonance imaging (MRI) has recently emerged as a clinical tool to image the lungs. This paper outlines the current technical aspects of MRI pulse sequences, radiofrequency (RF) coils and MRI system requirements needed for imaging the pulmonary parenchyma and vasculature. Lung MRI techniques are presented as a “technical toolkit”, from which MR protocols will be composed in the subsequent papers for comprehensive imaging of lung disease and function [1, 2].

This paper is pitched at MR scientists, technicians and radiologists who are interested in understanding and establishing lung MRI methods.

Images from 1.5 T and 3 T clinical scanners are used for illustration of the sequences and methods that are highlighted. This paper does not cover the methodology for hyperpolarised gas MRI of the lungs. Although highly attractive for the possibility of studying physiological processes such as lung ventilation, this technique requires sophisticated polarisation and multinuclear RF hardware, which is currently of limited access in routine clinical practice.

## Physical lung properties relevant to MRI

The physical properties of lung parenchyma are very different from those of tissue such as liver or brain. For MRI of the lung, two of these tissue properties are of highest importance: the low density and the susceptibility differences between tissue and air.

In healthy lungs the tissue density is 0.1 g/cm^3^, which is about tenfold lower than in other tissues. As the MR signal is directly proportional to the tissue proton density, even under perfect imaging conditions (i.e. neglecting relaxation effects), the MR signal of the lung is ten-times weaker than that of adjacent tissues. The low signal-to-noise ratio (SNR) makes structural proton MRI of the lung microstructure challenging: to increase the SNR, signal averaging can be employed, but this increases the image acquisition times beyond 10 min per data set, which would make the protocols unsuitable for clinical routine. SNR can be increased with larger voxel sizes; however, smaller lesions such as peripheral lung metastases might not be visible due to partial volume effects.

Oxygen in air is paramagnetic and tissue is diamagnetic, which leads to a bulk magnetic susceptibility difference (Δχ = 8 ppm) at lung–air interfaces. At each tissue interface the susceptibility difference forms a static local field gradient. The multiple microscopic surfaces presented by the airways and alveoli in the lungs thus create highly inhomogeneous local magnetic field gradients on a spatial scale smaller than the size of a typical imaging voxel (2–5 mm). These microscopic field gradients lead to a rapid dephasing in gradient echo imaging; this signal decay is typically described by an apparent transverse relaxation time T2*, which can be as short as 2 ms or less at B_0_ = 1.5 T. Thus, gradient echo MRI of lung parenchyma becomes highly challenging and requires pulse sequences with short echo times (TE < 1–2 ms).

**Fig. 1:**
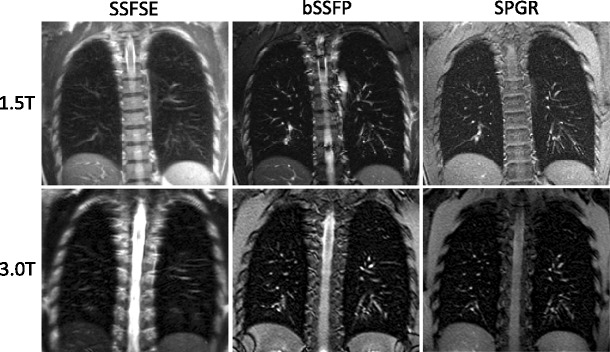
1.5 T vs 3 T anatomical image SSFSE, bSSFP and SPGR

As the magnetic field inhomogoneity increases with B_0_, even shorter T2* of about 0.5 ms are found at 3 T^1^. The expected SNR gain of 3 T over 1.5 T can often not be realised, because it requires that TE is shortened accordingly. With identical pulse sequences, a shorter TE can only be achieved if more powerful gradient systems are used, but current 3 T MR systems often utilise the same gradient units as high-end 1.5 T systems (Fig. 1).

Low-field MRI at B_0_ = 0.5 T or less has some potential advantages for lung MRI with respect to the magnetic inhomogeneity [3], and promising results have been demonstrated with low-field MRI as a non-ionising alternative to chest X-ray [4]. Low field strength does however require signal averaging to recover signal to noise, leading to longer imaging times, which is less attractive from a clinical perspective. The methods in this paper are focused on implementation at 1.5 T, as it represents a readily achievable optimal field strength for lung MRI.

**Fig. 2:**
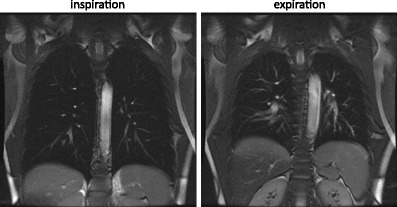
Balanced steady state free precession (bSSFP) images at full inspiration and full expiration

Most of the methods outlined here involve imaging during a breath-hold of typically less than 20 s duration. Breath-hold is achieved either in end-expiration or in full inspiration. The state of lung inflation plays a role, in that the signal intensity from the lung parenchyma is higher at expiration. During expiration the relative density of protons in a voxel of parenchyma is increased, and the bulk volume magnetic susceptibility difference is reduced as air is expelled. If, on the other hand, high contrast to noise between the pulmonary vessels or nodules is sought, imaging at full inspiration can provide a darker background (Fig. 2).

## Respiratory motion

In order to reliably acquire images at defined stages during respiration (e.g. in full inspiration), respiratory or spirometric triggering and gating can be used. Synchronisation of the image acquisition to the respiratory cycle requires the acquisition of a signal that is proportional to the respiratory state of the lung; in MRI this is achieved using either external hardware or the MR signal of the lung itself. The MR manufacturers often provide pneumatic bellows for this purpose, however, MR compatible pneumotachographs have also been used [5].

Alternatively, so-called navigator echoes can be applied to measure the breathing motion directly via MRI. Here, either a dedicated 90-180° spin echo excitation or a pencil beam readout is used to measure the MR signal orthogonal to the base of the diaphragm. A sub-second self-navigator scan can also be integrated into the imaging sequence [7] to provide the motion information. Thus, the motion of the diaphragm is detected and can be used for retrospective gating of scans during the free-breathing cycle [6]. A navigator signal can also be extracted from free-breathing non-gated two-dimensional (2D) lung images as long as the acquisition time is fast compared with the motion of the diaphragm.

## Pulse sequences

Modern MR systems are equipped with gradient systems that offer a gradient strength of 40 mT/m or more with slew rates of more than 200 mT/m/ms; thus, for a typical spatial resolution of 1–3 mm, very short echo times of TE < 1.5 ms can be realised with gradient echo sequences. At 1.5 T it is possible to acquire images that highlight regional fibrosis and parenchymal density. There are three basic sequences that have been found to be very effective for lung imaging. All share the common features of short TEs and short acquisition time needed when imaging the lungs and all can benefit from the judicious use of parallel imaging [8]. They can be used with or without respiratory gating, so that application during free-breathing or breath-hold is possible.

## Spoiled gradient echo (FLASH, SPGR, FFE)

The spoiled gradient echo sequence is conceptually the simplest imaging pulse sequence, and has been proven to be very robust in clinical practice. Essentially, the sequence acquires a gradient echo after RF excitation with a low flip angle RF pulse (α < 90°), and destroys the remaining magnetisation after the data acquisition (spoiling). The sequence is inherently T2*-weighted, and depending on TR and α either an additional spin density weighting or a T1-weighting can be achieved. As lung T2* values are short, a TE of 1 ms or less needs to be used to detect signal from lung parenchyma; furthermore, very low flip angles of α < 10° need to be applied to minimise T1 weighting due to the relatively long T1 of lung parenchyma (1,300 ms at 1.5 T). Use of an asymmetric echo (~30%) and partial Fourier reconstruction in the frequency encoding direction with a high bandwidth > 60 kHz ensure TEs ≤ 1 ms can be met.

The sequence has multiple applications in lung MRI; for anatomical imaging, it can be used for 2D and 3D acquisitions with or without fat suppression. Non-contrast-enhanced images are preferably acquired without fat suppression to facilitate the detection of lymph nodes surrounded by mediastinal fat. After injection of a T1-shortening contrast agent (e.g. Gd-DTPA) and with application of fat suppression, lymph nodes are emphasised within the suppressed background of the mediastinal fat [9].

Combination of the gradient echo sequence with parallel imaging methods and/or slice interpolation techniques (e.g. VIBE), enable full lung volume coverage with slices of 5 mm within short acquisition times of a breath-hold. Dynamic repetitive acquisitions at low resolution provide a robust means of assessment of lung wall motion for investigation of dynamic lung volumes [10], tumour motion for radiotherapy planning [11] and paradoxical diaphragmatic motion.

A heavily T1-weighted 3D spoiled gradient echo sequence with short TR and high α is the starting point for any contrast-enhanced structural imaging in the lung, with high flip angles an excellent suppression of non-enhancing anatomical regions can be achieved. Sequence parameters for pulmonary MR angiography and perfusion imaging will be dealt with in the section on contrast enhancement.

## Balanced steady state free precession (bSSFP—TrueFISP, FIESTA, BFE)

The bSSFP sequence is structurally very similar to the spoiled gradient echo sequence; however, as it does not spoil but instead re-focuses the transverse magnetisation at the end of each TR interval, it makes more efficient use of the available magnetisation. Within each TR all gradient dephasing is refocused (balanced), and by using RF pulses with alternating phases (i.e. the magnetisation is flipped back and forth), a highly coherent steady state can be achieved. Compared with the spoiled gradient echo sequence, the propagation of transverse coherence leads to a more complex contrast behaviour, and for short TR the contrast is proportional to the ratio T2/T1. Balanced SSFP sequences use higher flip angles (about 70°), which lead to a stronger signal, but also to higher specific absorption rate (SAR) values. Balanced SSFP sequences are susceptible to off-resonance and field inhomogeneities, which is manifested as so-called banding artefacts where one to several dark bands occur.

The fast bSSFP sequence has found many applications, in particular in cardiac MRI, but it can also be applied to anatomical lung imaging [12]. The similarity of the bSSFP sequence to the spoiled gradient echo sequence also means that short TE and TR can be realised so that it is well adapted for rapid imaging within the time window of a breath-hold. High readout bandwidths of 100 kHz are typically used to maximise magnetisation recycling and to minimise TR, which reduces the size of the off-resonance banding artefacts. The inherent T2/T1 contrast of the sequence [13] makes it highly effective for imaging blood and mucus with their long T2 when compared with tissue. Furthermore, a multi-slice or 3D bSSFP protocol can be used as a quick and contrast-free means of generating a pulmonary angiogram [14].

## Single-shot fast spin echo (RARE/HASTE, Turbo FSE)

The single-shot fast spin echo sequence (SSFSE) or RARE sequence [15] uses spin echoes (compared with FLASH and bSSFP, which utilise gradient echoes) and is thus conceptually different from the previous imaging sequences. To make the spin-echo acquisition suitable for rapid imaging, a train of spin echoes is created by repeated refocusing of the first spin echo. Thus, all lines in k-space are acquired in a single echo train.

**Fig. 3:**
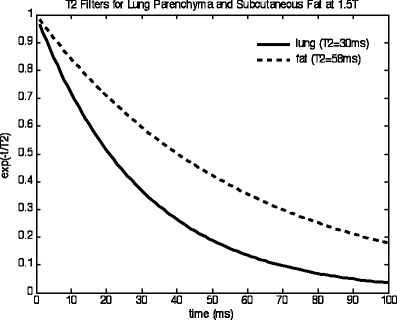
T2 filter for SSFSE

The SSFSE sequence is advantageous for lung imaging as the train of 180° pulses refocuses any field inhomogeneity so that the images become T2-weighted. The degree of T2-weighting is determined by the echo time of the central k-space line (effective TE), which can be adjusted through the choice of different k-space sampling patterns (Fig. 3).

Echo times of the order of the T2 of lung parenchyma (about 40 ms at 1.5 T) are still suitable for lung imaging. Compared with the sub-millisecond echo times needed for the gradient echo sequences such a longer TE is easier to realise; however, with an inter-echo spacing of about 4 ms, a typical echo train for an image with 192 phase encoding steps still amounts to 192 × 4 ms = 768 ms. In the later echoes of this echo train, the T2 decay will lead to significant signal reductions. The T2 decay in the PE direction also leads to a filter effect in k-space that manifests as blurring in the images. Thus, it is advantageous to reduce the number of measured k-space lines, which can effectively be achieved by combinations of half-Fourier encoding, rectangular FOV selection (e.g. in axial orientation, PE is chosen in anterior-posterior direction) and parallel imaging.

The large number of 180° pulses in the SSFSE echo train also leads to high amounts of energy deposition (high SAR levels), which make the use of SSFSE at 3 T difficult, as SAR increases with the square of the field strength.

Another option to reduce signal blurring in the PE direction is the segmentation of k-space. Therefore, not all k-space lines are acquired in one echo train, but only a fraction *N* is sampled, and the acquisition is repeated *N* times to cover the complete k-space. Segmentation significantly prolongs the acquisition time, but is often required when a spatial resolution of 1 mm or better is needed or respiratory-triggered acquisition is needed for uncooperative patients.

The SSFSE sequence can be combined with magnetisation preparation modules such as fat saturation, inversion recovery preparation or even diffusion weighting to impart additional contrast weighting to the images.

## RF coils and parallel imaging

**Fig. 4:**
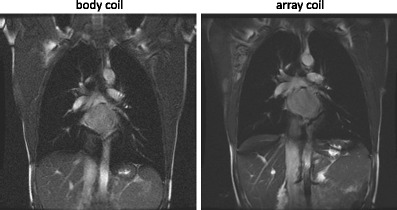
Body coil vs array coil

At a field strength of 1.5 T, the MR system’s body coil provides a relatively homogeneous transmit field, delivering a uniform flip angle over the FOV of the lung. At 3 T, flip angles in the thorax are no longer homogeneous, which is a consequence of the shorter RF wavelength. In principle, the body coil can be used as a receive coil for lung imaging, but a significantly higher SNR is achieved with a local receive coil array that is optimised for MRI of the thorax (Fig. 4). Typically, thoracic MR coils consist of an anterior flexible part and a posterior part that is embedded in the patient table. Depending on the number of available receiver channels, the coil arrays consist of 2–16 coil elements each. Elements are arranged in rows and columns to be able to accelerate the image acquisition in all directions using parallel imaging.

**Fig. 5:**
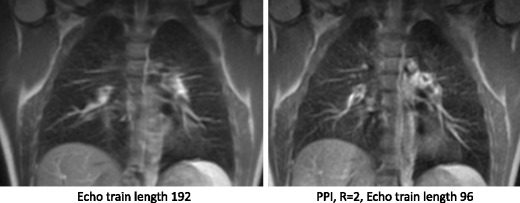
SSFSE with and without parallel imaging

Parallel imaging methods [8, 16] exploit the spatial sensitivity patterns of an array of coils to accelerate the image acquisition by factor *R*. Essentially, every coil in the array has an intrinsic spatial encoding of the MR signal, if the sensitivities of the coil elements are spatially discrete. Parallel imaging is useful in lung MRI, where faster acquisition times can reduce breath-hold durations and increase temporal resolution in time-resolved contrast studies. Parallel imaging is particularly useful in SSFSE imaging, as it shortens the echo train length [17] and the associated k-space filter thus reducing blurring (Fig. 5). At 3 T, parallel imaging can help reduce SAR of a SSFSE sequence, which can also allow a shorter echo time [18].

Parallel imaging should however be used carefully, because the acceleration achieved with parallel imaging comes at the price of a reduced SNR: an *R*-fold faster acquisition lowers the SNR by √*R* and g-factor noise amplification further reduces SNR in a spatially varying manner. As lung parenchyma SNR is intrinsically low, high acceleration factors of *R* > 4 as used in cardiac imaging are not achievable in lung imaging. As such, coils with a large number of elements are not essential for the parallel imaging of the lung (even though they can help to increase the local SNR without parallel imaging) and acceleration factors of *R* ≤ 2 in either phase-encode or slice selection/encode directions are appropriate.

An important detail of parallel imaging is the method of coil sensitivity calibration, which is needed to determine the sensitivity profiles of the coils for image reconstruction. As the lungs are prone to movement, a method with integrated or auto-calibration is preferable (e.g. GRAPPA [16], autoSENSE, FLEX). A sequence that requires a separate sensitivity scan is potentially prone to artefacts due to spatial mis-registration of calibration information with imaging data.

## Ultra-short echo time (UTE) imaging

**Fig. 6:**
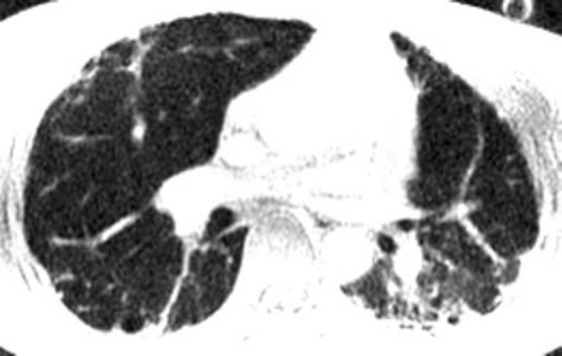
UTE at 1.5 T in a patient with interstitial lung disease

Originally proposed by Bergin et al. [19] for lung parenchyma imaging, ultra-short echo time sequences have recently become available that use radial k-space sampling to shorten the echo times well below 1 ms. At a TE of 100 μs and less, the short T2* of lung parenchyma is less of a constraint, and MR images with parenchymal sensitivity can be acquired. Two-dimensional UTE sequences [19] require twice the number of RF pulses in order to achieve slice selection, which doubles the acquisition time. Furthermore, the T2* decay of the parenchymal signal introduces a k-space filter in the frequency encode direction (akin to that experienced in the phase encode direction in SSFSE imaging) which limits the achievable spatial resolution well below the nominal one. Nevertheless, UTE imaging shows promise for lung MRI, because a proton density contrast can be achieved that resembles that of CT. Furthermore, this pulse sequence enables visualisation of parenchymal lung disease, which has so far been impossible with MRI (Fig. 6).

## Contrast enhancement

**Fig. 7:**
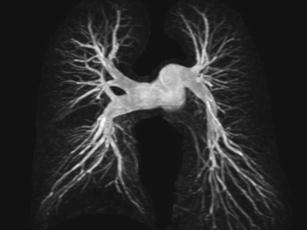
Breath-hold CE pulmonary MRA

High-quality pulmonary angiograms can be obtained at breath-hold with injection of T1-shortening contrast agents such as Gd-DTPA using heavily T1-weighted 3D GRE sequences [20]. The pulse sequence of choice for high-resolution 3D pulmonary MRA is a spoiled gradient echo (FLASH, SPGR, FFE) with the shortest possible TR and TE and high readout bandwidth. Modest parallel imaging can improve the spatial resolution for a given breath-hold duration [21] (Fig. 7).

For an adult patient, the typical imaging parameters for a coronal 3D data set are: TR = 2.5-3 ms, TE = 1.0-1.5 ms, α = 30-40°, matrix: 40 × 192 × 256, FOV: 460 mm, parallel imaging factor *R* = 2. Centric elliptic phase encoding [22], with the scan acquisition starting at peak enhancement ensures maximum SNR and optimal separation of arterial and venous phases. Typical contrast doses used are for example 0.2 mmol/kg body weight Gd-BT-DO3A followed by a 20 ml saline flush injected at 5 ml/s.

**Fig. 8:**
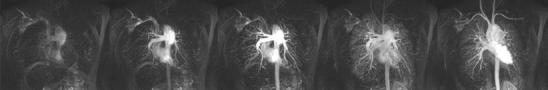
Time-resolved bolus passage TRICKS data set

The time from injection of the contrast agent to start of acquisition can be optimised with a time-resolved bolus-training scan following injection of a 1-ml bolus of contrast agent. Alternatively, a time-resolved 3D acquisition with view sharing such as a TRICKS sequence [23] can be used, which additionally delineates the arterial and venous regional haemodynamics, regional perfusion defects and cardiac shunts. With TRICKS, k-space data are shared between successive data sets, which leads to minor temporal interpolation artefacts—thus, TRICKS data should be used with care when a rapid signal change is observed (as, for example, during bolus arrival) (Fig. 8).

Quantitative T1-weighted perfusion images can be obtained by using a time-resolved low spatial resolution version of the sequences described above. Here, typical imaging parameters would be: TR = 2.0-2.5 ms, TE = 0.8-1.0 ms, α = 30-40°, matrix: 32 × 96 × 128, FOV: 460 mm.

**Fig. 9:**
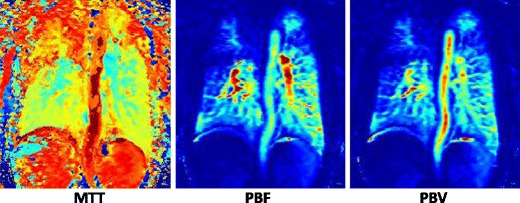
Parametric maps of perfusion

With a temporal resolution of better than 1 s per 3D data set this technique allows the generation of contrast passage kinetics curves and parametric maps of regional blood flow, volume and transit times. Again, parallel imaging and view sharing can enhance the temporal resolution. The contrast agent dose should be less than is needed for the pulmonary angiogram; for example, 0.05 mmol/kg Gd-BT-DO3A + 20 ml saline flush at 5 ml/s provides robust results in patients with pulmonary vascular disease. If fully quantitative maps of pulmonary blood volume (PBV), blood flow (PBF) and transit time (MTT) are sought, a deconvolution with an arterial input function (AIF) measured in the larger arteries is required. For this, weaker doses still may be needed to ensure a linear signal response between contrast concentration and the signal of the AIF due to T1-weighting [24] (Fig. 9).

## Functional sensitivity of proton lung MRI

The basic pulse sequences outlined above can be further sensitised to physiological processes. Their signal can be modulated by respiration, blood flow, and inhalation of oxygen to give functional information. Techniques for functional lung MRI are briefly outlined.

## Oxygen-enhanced ^1^H MRI

**Fig. 10:**
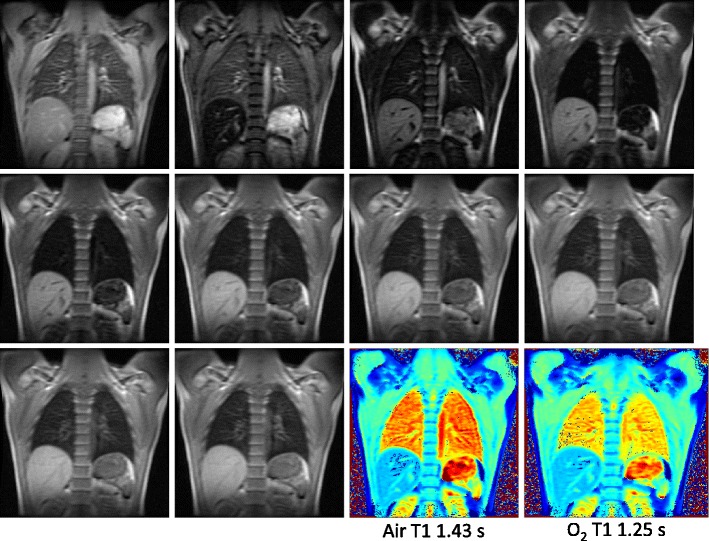
A series of images acquired during inversion recovery at different inversion times with a Look-Locker sequence and the resulting T1 maps obtained breathing air and oxygen

When a subject breathes pure oxygen instead of room air, the paramagnetic oxygen shortens the T1 of the blood, plasma and tissue in the lungs. This T1-shortening leads to a signal enhancement, which can be quantified with parametric maps with an inherent ventilation and perfusion weighting. Two strategies for ventilation and perfusion imaging have been proposed:

The first technique is semi-quantitative and utilises an inversion-recovery HASTE sequence. The 2D HASTE readout uses a short echo time to visualise the lung parenchyma; however, prior to the data acquisition a non-selective inversion pulse is applied followed by an inversion delay TI to impart an additional T1 contrast on the HASTE image. A global inversion pulse should be used rather than a slice selective one to avoid inflow effects of blood with a different magnetisation history [25]. The TI is chosen so that the signal from the perfused pulmonary vascular bed is nulled when breathing room air (at the T1 of the lung parenchyma 1,100–1,300 ms at 1.5 T [26] , TI = 0.69 × T1 = 700–900 ms). Pure oxygen is then administered via a tight-fitting face mask, which reduces the mean T1 of the lung plasma and tissue, and a signal enhancement is seen in the IR-HASTE images [27]. The technique is implicitly ventilation- and perfusion-weighted and thus represents an indirect way of measuring both aspects of lung function. The method can be made more quantitative with T1 mapping which uses the inversion pulse as IR-HASTE, but a series of low flip angle FLASH images (Look-Locker sequence [28]). This allows quantitative inferal of lung tissue/plasma partial pressure of oxygen (p0_2_) to be made [29, 30]. A series of Look-Locker images is shown in Fig. 10, together with the T1 maps of the lung for both room air and pure oxygen breathing.

An alternative method uses a bSSFP sequence to repeatedly acquire images from the same slice [31]. The time course of the signal is then analysed by Fourier decomposition giving images which are weighted by lung perfusion at a frequency of around 1 Hz (the pulse rate) and images which are weighted at a lower frequency (~0.2 Hz) by respiratory motion. Thus, ventilation and perfusion-weighted images can be derived from that slice [32]. To implement the bSSFP method, high SNR is paramount, which can be achieved using centric encoding and acquisition during the transient steady state and a magnetisation flip back pulse after each acquisition.
